# Second-Generation Digital Health Platforms: Placing the Patient at the Center and Focusing on Clinical Outcomes

**DOI:** 10.3389/fdgth.2020.569178

**Published:** 2020-12-03

**Authors:** Yaron Ilan

**Affiliations:** Department of Medicine, Hadassah Hebrew University Medical Center, Jerusalem, Israel

**Keywords:** precision medicine, artificial intelligence, algorithms, variability, complex systems

## Abstract

Artificial intelligence (AI) digital health systems have drawn much attention over the last decade. However, their implementation into medical practice occurs at a much slower pace than expected. This paper reviews some of the achievements of first-generation AI systems, and the barriers facing their implementation into medical practice. The development of second-generation AI systems is discussed with a focus on overcoming some of these obstacles. Second-generation systems are aimed at focusing on a single subject and on improving patients' clinical outcomes. A personalized closed-loop system designed to improve end-organ function and the patient's response to chronic therapies is presented. The system introduces a platform which implements a personalized therapeutic regimen and introduces quantifiable individualized-variability patterns into its algorithm. The platform is designed to achieve a clinically meaningful endpoint by ensuring that chronic therapies will have sustainable effect while overcoming compensatory mechanisms associated with disease progression and drug resistance. Second-generation systems are expected to assist patients and providers in adopting and implementing of these systems into everyday care.

## Introduction

The use of artificial intelligence (AI)-based systems in medicine has drawn much attention over the last decade (1). However, high expectations have not always been met by reality. Various obstacles have arisen, blocking AI platforms' validation and implementation (2). AI-based algorithm applications have been slower to spread in medical practice than anticipated. Obstacles include difficulty in achieving adequate algorithm stability, mainly due to difficulties associated with big data analysis, and also the failure of these methods to contribute to noteworthy clinical effects. The need for improved clinical outcomes, is a barrier to the adoption of AI by patients and healthcare providers (3–7).

The AI “quadruple aim” of improving care, improving overall population health, reducing healthcare costs, and improving the work life of healthcare providers, is still an unreachable objective for the majority of first-generation systems (8). These platforms were designed to promote the 4P model of medicine: Predictive, Preventive, Personalized, and Participatory, providing patient autonomy (9). This paper reviews some achievements of first-generation systems used over the last decade, and discusses difficulties of implementing them in practice. The development of second-generation platforms in the upcoming years may overcome some of these hurdles. Second-generation systems aim at focusing on a single subject and on improving clinically meaningful endpoints and clinical outcomes. This makes their implementation in everyday care more viable. Establishment of personalized closed-loop platforms, which improve organ function and response to chronic therapies, is presented, with the introduction of individualized patterns of variability discussed as an example application for second-generation AI.

### Benefits of First-Generation Artificial Intelligence Platforms in Clinical Practice

AI has been proposed to serve in augmented medicine platforms to improve different aspects of clinical practice. However, AI's real world utilization in clinical practice is limited (1, 10–14). Most algorithms do not inevitably result in better outcomes (10). The term “AI chasm” is sometimes used, and this term suggests that the improved accuracy sought by most platforms does not necessarily represent better clinical efficacy (15). Unsurprisingly, a lack of clear beneficial effects is a major obstacle to adoption of AI in clinical medicine.

First-generation AI systems developed over the last decade have largely focused on clinical decision making through big data analysis as a way to generate evidence-based information. These include supervised machine learning, where the algorithm based on a labeled dataset, provides a model that the algorithm can use to determine its accuracy on training data (16); An unsupervised modeling which provides unlabeled data that the algorithm attempts to fit by extracting patterns on its own (17); recommender systems that seeks to predict the preference a user would give to an item (18); expert systems which attempt to match the decision-making ability of a human expert (19); natural language processing which process and analyze large amounts of natural language data (20); computer vision which analyzes data from digital images or videos (21); and expert-guided feature extraction schemes, where derived features are based on an initial set of measured data for facilitating subsequent learning and generalization steps (22, 23).

Machine learning (ML)-based techniques and neural networks can rapidly process numerous inputs. Thus, they can discover complex associations which are not easily analyzed by regular equations, which assists in reaching better clinical conclusions (1, 24–26). Electronic medical records (EMR) and digital databases are used for these purposes (27–29). Precision medicine-based AI is aimed at optimizing diagnostic pathways, therapeutic interventions, and prognoses using large datasets comprising individual genes, other phenotypic parameters, and environmental measures. The algorithms are largely designed to tailor their outputs on an individual basis (30).

A relatively limited number of settings in clinical practice currently benefit from the application of first-generation systems. A review of 450 published AI articles highlighted four areas: risk stratification of populations at risk, clinical decision support, disease screening, and tools for patient self-management (31). First-generation AI can reduce variations in clinical practice, improve efficacy and reduce medical errors (32). It has shown advantages when extracting and analyzing a large volume of data from EMR (33), developing risk scores (34), predicting mortality, readmission risks, prolonged length of stay, and failure to attend appointments (35, 36), and for summarizing doctor-patient consultations (10, 37).

First-generation systems have also been found useful for detection of atrial fibrillation and epilepsy seizures (11). These methods also showed some degree of success when interpreting phenotype and genomic data for genomics-based diagnosis (38), and for assessment of cancer risk (39–42). Benefits were also documented for identifying cancerous skin lesions (43–45), diagnosing cancer in computational histopathology (46), interpreting retinal imaging (47–51), detecting arrhythmias (52, 53), polyp detection from colonoscopy (54), identifying genetic conditions from facial appearance (10, 55), predicting acute kidney injury (56), detecting epilepsy (57), process images from endoscopy (58, 59), and predicting outcomes of gastrointestinal bleeding (58, 59). Some advantages were likewise shown for decision making in sepsis management (60), and in pharmacogenomics (61, 62). Even self-monitoring of blood glucose can be facilitated by AI (63), helping to improve blood glucose control, reduce hypoglycemic episodes, and reduce diabetes-related complications (31). The resulting “do it yourself” automated insulin delivery for diabetics exemplifies democratization of medicine by providing privacy, intelligent computing, and sharing of information (64).

In the field of imaging, AI showed benefits for interpreting chest radiographs (65–67), detecting cancer in mammograms (68), analyzing computer tomography scans (69, 70), identifying brain tumors in magnetic resonance images (71), and predicting the development of Alzheimer's disease from positron emission tomography (72). AI can also assist radiologists by identifying abnormal or negative exams. Centralized interpretation of chest radiographs can compensate for a shortage of radiologists in under-resourced areas (73). Web-based applications can be used to augment psychiatric services (74). Smartphone-based applications are used to improve adherence to medications, and to monitor heart rate, activity levels, sleep levels, and electrocardiogram (ECG) tracings (75, 76). Algorithms outperformed cardiologists in diagnosing heart attacks by ECG (77), and outperformed dermatologists in classifying skin lesions as benign or malignant, due to their rapid learning from multiple cases (43). These algorithms also performed better than experts in analyzing pulmonary tuberculosis in chest radiographs (78).

In addition to these applications, AI can support preventative medicine by determining the risks for complications that would warrant intervention. Patients identified as low risk receive reassurance while high-risk patients are referred for intervention (1). AI is used for precision therapeutics, for repurposing drugs, and for drug development, by analyzing large volumes of data (30). Several algorithms have yielded unanticipated findings, such as predicting breast cancer prognosis using stromal cells (79), predicting cardiovascular risks using fundus photographs (80), identifying atrial fibrillation from ECGs acquired during sinus rhythm (53), and earlier diagnosis of dementia using retinal imaging (10, 81).

Many of the studies described above were retrospective and used historical data for training the algorithms. However, prospective studies are required to prove software's utility in real world. Prospective studies have shown limited benefit in diabetic retinopathy grading (82–84), detection of breast cancer metastases in lymph node biopsies (85), wrist fracture detection (86), colonic polyp detection (87), and detection of atrial fibrillation in Apple watch users (10, 88). Results have been shown when predicting risk of certain cancers (39, 89, 90) and for cardiovascular diseases (80, 91). Lesser results have been noted for epilepsy, and for neurodevelopmental disorders, including intellectual disability and autism spectrum disorder (30, 92, 93).

Only a few AI-based randomized controlled trials (RCTs) have been conducted to date. These include a trial to detect childhood cataracts which showed lower performance compared to senior clinicians (94), a single-blinded RCT that demonstrated reduced blind-spot rate in gastroscopy (95), an open non-blinded trial for polyp detection by colonoscopy which showed improved detection rate of diminutive adenomas and hyperplastic polyps (96), a trial to detect acute neurologic events (97), and an open trial for analyzing cardiotocographs during labor that showed no improvement in clinical outcomes (98).

Overall, the data have shown some benefits of first-generation platforms for certain tasks with a defined input and a binary output (1).

### Obstacles Faced by First-Generation AI Systems Associated With Limited Penetration Into Clinical Practice

The data used by the first-generation systems are a major cause of difficulties. Large databases sometimes lack well-structured training sets that are stable over time. Many of the current algorithms lack the ability to make clinically relevant associations when used for analysis of EMR, physicians' notes, natural language processing (NLP), identification of patterns within datasets, or attempts to associate patients' phenotypes and genotypic markers (30, 99). Sources may suffer from the absence of a unifying data format across centers (100). Batch effect technology biases are obstacles in analyzing population scale omics and imaging data (101–103). Inadvertent discriminatory bias, inapplicability outside of training domains, and propagation of unintentional biases in clinical practice all affect outputs. Moreover, a lack of valid interpretability and of reliable measures for model confidence affects accuracy (1, 10). Input data is produced within non-stationary settings with shifting patient populations, and changes can occur in clinical practices. Dataset shifts, which happens when test and training inputs and outputs have different distributions, commonly occur when these realities are disregarded (10, 104).

The retrospective designs of most studies and their limited sample sizes lead to selection bias and overfitting. Selection bias is introduced by selection of data in a way that proper randomization is not achieved, thereby ensuring that the sample obtained is not representative. Overfitting is a modeling error that occurs when a function is too closely fit to a limited set of data points (10, 58, 105). Small sample sizes also lead to marked variability in predicting risk, such as for cardiovascular disease, decline of glomerular filtration rate in polycystic kidney disease, and risk of progressive IgA nephropathy (106, 107). Using fitting confounders rather than true signals, unintentional fitting of confounders, or “over-fitting” may impact the accuracy of output. Accidentally fitting confounders vs. true signals or simply using whatever signals are available to achieve the best performance in a dataset are ML practices associated with bias (10). AI may give undue importance to spurious correlations within past data, as exemplified by a failure to predict seasonal prevalence of influenza (108).

Data-related obstacles such as differences between sites' EMR, laboratory equipment, coding definitions, and clinical practices undermine the reliability and generalizability of medical AI systems (10). Comparing algorithms across studies is a major difficulty, as they often use different methodologies on diverse populations. Discriminatory bias can lead to inaccuracies when working with minority subgroups, as AI can disproportionate affect groups disadvantaged by factors such as race, gender, and socioeconomics (10, 68, 109–113). Implementing generalizations is challenging across new populations where specificity at a fixed operating point varies across independent datasets (65, 70). For instance, one system would classify a skin lesion as malignant if an image included a ruler, which correlated with an increased likelihood of diagnosing a cancerous lesion (43). Another interpreted surgical skin markings falsely as evidence of increased melanoma probability scores (114). A third system classified images of malignant moles, but was trained on fair skinned patients, and underperformed on images of lesions in dark skin (43, 115, 116).

A lack of validation sets is also a major drawback for some of the platforms used (10, 117, 118). A recent review found that only 6% of 516 eligible published AI studies performed external validation (109). Real-world clinical performance necessitates external validation. By testing the algorithms on properly sized datasets collected from multiple institutions, it can be ensured that variations in patient demographics and disease parameters are represented in the model (113).

AI can examine multidimensional data and identify patterns that capture multiple parameters which determine disease progress and response to therapy. These include genotype, metabolism, drug pharmacokinetics, disease characteristics, comorbidities, and environmental factors (30). Individualizing phenotypic or genotypic patterns requires high-performance computing for processing multidimensional datasets (30, 49, 55, 119–122). Despite this, high phenotype overlap between different disorders, and phenotypic variability between individuals with the same diagnosis, may lead to misdiagnosis (112, 123, 124). Quantifying risks from patterns of genomic variation involves marked heterogeneity, variable penetrance of risk variants, and environmental factors (30, 92, 112, 125–129). Numerous penetrant variants are linked with more than one clinical manifestation, while several diagnoses are described by variable presentations (30, 122, 130). Inability to address gene-gene interactions involving more than few genes (131, 132), and overlap between gene lists and/or protein/co-expression networks for different diseases, reduce diagnostic accuracy (125, 128). A high degree of overlap in common-SNP based genetic etiology and patterns of differentially expressed genes are associated with a low diagnostic accuracy (133, 134). To make matters worse, unknown variants' penetrance can be a hurdle in classifying mutations according to clinical relevance. While low penetrance is common, high penetrance variants are not unheard of. Most variants are non-coding, and defining their pathogenicity requires multidimensional data analysis (3, 105, 121). Many syndromes are diagnosed by a threshold number of symptoms and clinical history. The availability of data varies between subjects. Lack of concordance among experts, and dynamicity of symptoms which change over time, complicate the use of AI to reach accurate clinical results (135).

Many studies comparing the efficiency or accuracy of AI to that of clinicians can be shown to have an unreliable design, to be hard to replicate, or to lack validation (11, 112, 136, 137). A flowchart approach combines history taking and symptoms for diagnosis and requires large amounts of data. A database approach is based on the use of ML or pattern recognition to identify groups of symptoms or certain clinical image patterns (76). But since numerous signs can only be observed by a physician, many of these platforms are inferior to humans. AI-based software has been claimed as improving interpretation of pulmonary function tests, but the study was criticized for the AI scoring lower than average scores provided by clinicians (138). Computer-assisted diagnosis in screening mammography showed suboptimal positive predictive values, false positives, sensitivity, and specificity values (139–141). The study's algorithm, developed by reviewing 640,000 digital mammograms, could only reach a specificity, sensitivity, and area under the receiver operator curve similar to those of the bottom 10% of radiologists (142). When searching for a diagnostic of serious disease, AI can easily identify trivial data relationships which may be of no clinical significance, and therefore worsen over-diagnosis and overtreatment (143).

Many AI systems fail to explain the decision-making algorithm in an understandable way (10). Concerns regarding unacceptable results, the risk of unquantified biases that may prove difficult to identify, and the potential to use inappropriate confounding variables, all make it extremely desirable that the AI be explicable (10). AI trained by high volume data may recognize patterns that are not observable to humans (144), and which may be difficult for humans to deduce by observation. However, the trade-off between performance and explicability implies that the best performing models will often be the least explicable, whereas linear regression or decision tree models, which show poorer performance, are more explicable (10, 73, 113, 119, 145). Furthermore, increased administrative burdens associated with EMR and the lack of a legal framework defining liability when adopting or rejecting algorithm recommendations present additional barriers to the adoption of medical AI (11, 146–149).

Taken together, these examples highlight some of the barriers which underlie medical AI's limited accuracy and inability to achieve significant, reliable clinical impacts. These limitations help to explain the low penetration of first-generation AI into clinical practice.

### Second-Generation Systems Provide the “5th P”: Focus on Improving the Clinical Condition of a Single Patient

Healthcare applications of AI research have received extensive investments in recent last year's (76, 150). However, the stereotypical “Silicon Valley mindset” calling for engineers to “move fast and break things” is clearly inappropriate for applying AI to healthcare (143, 151). Currently most tasks performed by these platforms are limited, leaving the primary responsibility of patient management with a human doctor (1). First generation systems were designed to promote the 4P model of medicine: Predictive, Preventive, Personalized, and Participatory, providing patient autonomy (9). Second-generation AI systems are anticipated to add the “5th P:” Progress, an improvement of a clinically meaningful endpoint in a subject-tailored manner. Rather than analyzing data for assisting in diagnosis, prediction, or tailoring therapy, second-generation platforms focus on improving biological processes. Several fundamental changes needed for achievement of the “5th P” are described below.

#### Aiming at Clinical Outcomes

A focus on clinically meaningful endpoints and clinical outcomes that have a quantifiable benefit, and on improving patient outcomes, is expected to assist in adoption of these new platforms by patients and caregivers (10). These systems, when designed to assist in response to interventions, will need to generate outputs based on quantified clinical responses. If a clinical benefit is not achieved, the likelihood of these algorithms being implemented is low (30).

#### Improving Organ Function and Response to Therapies

For second- generation AI to improve quantifiable symptomatic or laboratory endpoints, it will have to improve organ function, mental healthcare, and response to drugs. The algorithms ought to improve biological systems, but not by attempting to change them, nor by pushing them in the opposite direction from the one they are tilted toward by a disease. Their goal should be “getting the train back on track,” independently of which direction the system has been skewed (152, 153).

#### Controlling for the Dynamic Nature of Host and Disease

Biological systems are dynamic by nature. Subjects, disease processes, and responses to therapies continually change. Chronic diseases move along a dynamic trajectory, creating a challenge of unpredictable progression, which is often disregarded by first-generation AI (30). The internal and external parameters that determine the progression of chronic disease and host response vary constantly. These require constant adaptation of therapeutic regimens (154). Furthermore, many therapies do not show efficacy or loss of response until considerable time has passed. Subjects may remain essentially untreated for several months in cases where reaching an effect requires several attempts. For many drugs, secondary loss of response occurs following an initial benefit (155, 156). Second-generation AI systems which are designed to improve response to therapies, must therefore facilitate analyzing inter-subject and intra-subject variabilities in response to therapies over time (157–160). The dynamicity of biological systems requires algorithms to implement continual, periodic system-wide updates and identification of performance deficits over time in an individual subject (10).

#### Overcoming Big Data Challenges by Implementing an n = 1 Concept

Most first-generation AI systems extract data from large databases, and artificially impose a rigid “one for all” algorithm on all subjects. Attempts to constantly amend treatment regimens based on big data analysis might be irrelevant for an individual patient. Imposing a “close to optimal” fit on all subjects does not resolve difficulties associated with dynamicity and the inherent variability of biological systems. Second-generation AI systems must therefore focus on a single patient as the epicenter of an algorithm and to adapt their output in a timely manner. They are required to continually respond to feedback in an individualized manner while generating an insightful database that can be used to further improve the algorithm for other patients.

These platforms may not require a large volume of high-quality data. Second-generation systems are expected to be able to function based on input from a single patient (158–160). Conventional ML systems developed to analyze massive datasets are not analogous to the way brains perform. The brain learns by analyzing data within a certain context. It does not need to watch a thousand airplanes to differentiate an airplane from a bird. This difference in approach is problematic when trying to achieve good outcomes for individual patients. Generalizing from large datasets to a single patient is unsuccessful in many cases, due to a large heterogeneity among subjects, and to ongoing individualized changes in disease triggers and host responses. The *n* = 1 concept can be implemented into second-generation platforms by focusing on dynamicity of disease and response to intervention in a single patient. The multiple host, disease, and environment-related variables learned from big datasets can be implemented into a single subject-based algorithm that analyzes input from, and generates output to, that subject (158–160).

In addition to the above four requirements for second-generation platforms, several additional parameters must be considered. These platforms should improve patient care, cost-effectiveness, and workflow for everyday clinical practice in terms of decision making and the physicians' workload (1, 161). They must use metrics which are clinically important and intuitive to clinicians (10). Reducing bias, using explainable approaches, and using common statistical methods are also desirable, as they improve transparency and trust, and encourage adoption by clinicians (10, 162–164).

### Establishment of a Second-Generation AI System: Implementing Individualized Signatures of Variability for Promoting a Subject-Tailored Improved Response to Therapy

#### Variability Characterizes Biological Systems

Algorithms that implement natural patterns into biological systems are being evaluated (160, 165–169). Rather than enforcing an artificial change on a biological process, such algorithms are designed to make use of rules that are inherent to the system.

Biological systems are characterized by a high degree of variability and randomness. The rules by which these systems behave are not fixed and continually change over time. Their dynamicity, and the multiple variables that continually affect these biological laws, stand in contrast to the laws found in physics. In physics, laws are regulated by more constant, more predictable rules (170–173). In contrast to randomness in physics, where only a fixed range of possibilities exists, in biology this range is itself variable, and sometimes even randomly constituted (174–177). Some may argue that biological systems may be less random and appear random when represented by linear or additive models. While these theories are more easily explainable when represented as complex systems, they do not rule out the option of some degree of intrinsic variability (178).

The term “randomness” in biological systems is thus used with several meanings. In some studies, it describes disorder in a thermodynamic sense; others use it when referring to a high degree of complexity of a process (153, 160, 165, 179, 180). Randomness is used to describe noisy or stochastic behavior of a system, or when referring to unpredictability of its structure and behavior (166, 174, 180–183). Randomness has evolutionary associations, wherein variation and probability are linked to the adaptability of a system (184, 185). Darwinian evolutionary processes are partially ascribed to an ability to adapt to unpredictable environmental changes. Neo-Darwinism suggests “blind chance” as an origin of variation. Spontaneous and induced mechanisms of phenotypic adaptation indicate a role for chance in compensatory processes (185–190). Chaos theory describes the process of disorder arising in deterministic systems that are sensitive to initial conditions, increasing the unpredictability of the systems (191).

The noisy or stochastic behavior of biological systems characterizes their dynamic and adaptable behavior in response to internal and external triggers (169, 192). Intrinsic stochasticity has been described for numerous intracellular pathways (181, 193). Organisms are dissimilar to each other, and undergo unpredictable changes affected by multiple irregular variables (158–160, 194, 195). Intra-subject and inter-subject biological variability is observed at every level from cellular organelles to whole organs (160, 196–201).

The Crick and Watson interpretation of molecular biology, influenced by quantum mechanics, holds that functional order is a result of order at the molecular level, where information passes from DNA to proteins (202, 203). However, DNA-dependent processes do not assure functionality at molecular levels. Proper function involves multiple correlations between processes at a higher level. As Schrödinger once argued for the physics of subatomic particles, it has been proposed that molecules in an organism cannot avoid stochasticity (203–206). Variability has been described in nucleotide substitutions in DNA sequence (207), and when referring to evolving ancestral DNA sequences, as they begin to take different forms in populations of the same species (208). Random genetic drift is an example of fluctuating gene variants in a population (209). Variability has been shown for cell proliferation and death decisions, evolving from heterogeneity in founder cells (210, 211). Phenotype variability has been described for biomarkers on lymphocyte subpopulations. Antibody response to pathogens comprises expansion of antigen-specific cells and involves stochastic competition among competing cell fates or deterministic cell fate decisions (212). A high rate of variability in generating regulatory T cells has been observed in immune disease responses (213). *Ex vivo* cytokine release tests have manifested high inter-individual and intra-group variability (214).

#### Variability Contributes to Proper Function of Biological Systems

Variability should be viewed as a property of causal processes that contribute to proper functions of systems (158, 159, 196). Keeping “steadiness” is not a mandatory objective of physiologic control under all conditions. It can prohibit normal adaptation to ongoing changes (158–160, 194, 195). Non-linear systems can function far from equilibrium, and their dynamicity is part of the plasticity required for optimizing their function (203, 215–217). Biological variability is desirable for the evolutionary dynamics that contribute to stability through adaptation (153, 160, 180, 218, 219). It should be regarded as a method used for generating “new order,” assisting in overcoming errors in assembly and functions (166, 220). For stochastic cellular processes such as single-cell responses, randomness-based modeling improves deterministic models (221–223). Additional examples include gatherings of cells within heterogeneous spaces and random control of associations of biomolecules, which lead to an array of synchronized functions, including transcription, translation, ribosome biogenesis, chromosome replication, and metabolism (160, 196, 224–226). The dynamic instability of microtubules likewise demonstrates that variability is required for their normal structure and function (196, 227).

#### An Advanced Model for Disease Involving Loss of Variability

Since variability characterizes the normal function of systems, health can be perceived as a continual adaptation which requires constant adjustments. Appearance of “order” in a system that is inherently disordered is associated with the occurrence of a disease, representing rigid dysfunction of a system (228–231). Loss of complexity in diseased states is related to a reduced ability to adapt to changes, and may underlie chronic illnesses and aging (232–234).

#### Irregularity in Response to Disease-Inducing Triggers and to Therapies

Variability occurs between subjects and within the same subject in the response triggers that induce a disease. An example of personalized response to disease-inducing triggers has been shown in a concanavalin A (ConA)-induced immune-hepatitis model. Marked variability in individualized responses to a disease-trigger was documented under similar conditions (180).

Similarly, marked inter-subject variability in the response to immunomodulatory therapies has been shown by marked differences in degree of alleviation of the immune-mediated liver injury. A personalized response has been documented for the effect of therapies on cytokine levels and on expression of cell epitopes on lymphocyte subsets (180). A physiological lattice model for liver metabolism of drugs has shown that variability of sinusoidal structure underlies variances in drug response in individual hepatic veins at different times, suggesting that individual ports react differently in response to drugs (235). In humans, unpredictability has been described when discussing the response to chronic medications with high rate of loss of response over time, and in some cases paradoxical exacerbation of diseases. Inter and intra-patient variability in drug serum levels between days has been shown (236, 237).

Variability in response to medications among subjects is commonly attributed to pharmacogenomics and pharmacodynamics-based drug metabolism (237–240). However, heterogeneity cannot be solely attributed to these mechanisms (241). The unpredictability of responses to drugs is partly due to the changing dynamic rules by which biological systems function. Complex intracellular drug-target interactions which are not defined solely by simple diffusion and intrinsic chemical reactions, and that are beyond “simple” pharmacodynamics, have been demonstrated. Non-specific interactions can slow the incorporation kinetics of DNA-binding drugs, and can be responsible for irregular drug diffusion in cells (241).

#### Loss of Response to Chronic Therapies

Individual resistance to drugs is highly variable and results from multiple genetic and environmental factors. This implies that deterministic equations are unsuitable for examining dose-response relations (242). For instance, a secondary loss of response to anti-tumor necrosis alpha (TNF) occurs in 25–61% of patients with rheumatoid arthritis following an initial effect (243–245). More generally, cancer drug resistance associated with patients' genetic background or acquired by tumors is common in patients with malignancies (246, 247). Resistance to anti-depressant medications occurs in a third of patients (248). Similarly, about a third of epileptics develop resistance to anti-epileptic agents (249).

#### Implementing Personalized Variability Patterns for Improving Function of Biological Systems and Response to Drugs

Regular fixed therapeutic regimens may not be compatible with physiological variabilities in biology, and can underlie loss of response to chronic drugs (158–160, 250, 251). Fixed regimens are incompatible with the variable trajectories which underlie the pathogenesis of diseases and compensatory responses to therapies (215). Introducing variability into therapeutic regimens can improve response to drugs. Dose escalations, reductions, and intermittent dosing with drug holidays can exert clinical benefits while minimizing adverse effects (252–256). For instance, in a prospective trial of patients with inflammatory bowel disease treated with anti-TNFs, loss of clinical response was observed in 36% of patients on fixed dosing compared with only 13% in subjects treated using a (de-) escalation dashboard (257).

The variability which characterizes properly functioning biological systems, their response to disease-inducing triggers, and their response to therapies, along with real-world data showing beneficial effects of drug holidays and dose escalation/reduction, supports implementing variability-based regimens. This holds promise for improvement of organ function and response to therapies (158, 159, 165, 180, 258–265). Introducing variability into systems increases their degree of complexity and improves their function (160, 266). The resulting variability pattern tracks a similar trajectory to that used by the body while responding to disease-triggers and to therapies. This is expected to improve response to interventions under unpredictable conditions (158–160, 165, 166).

It has been proposed that the type and magnitude of variability in a biological system should ideally be personalized (158, 159). It has been suggested that continuously quantifying individualized variability patterns and implementing them into algorithms enables personalized and dynamic tailoring of therapeutic regimens. Patterns of variability can be quantified from genetic profiling, immune testing, chronotherapy measures, heart rate variability, and additional host and disease-related variability signatures (162, 163, 170, 171). This type of improved precision medicine is compatible with the principles according to which diseases and hosts behave. Rather than imposing a rigid artificial regimen, algorithms continuously adapt to individual changes in disease and the patient's responses to interventions (158–160, 166). Therefore, a patient-tailored approach based on individualized variability patterns implemented into AI systems is expected to improve the efficiency and sustainability of the beneficial effects of chronic therapies.

#### A Continuously Adapting Individualized-Variability Based Closed-Feedback Loops for Ensuring Plasticity and Improved Function of a Biological System

The establishment of the second-generation AI systems described herein is performed in steps (267). In the first step, the effect of introducing variability into therapeutic regimens of patients who have partially or completely lost their response to chronic medications should be evaluated. These algorithms have embedded pseudo-random number generators which introduce variability in times of administration and dosages within an approved range. These pseudo-random number generators function within regulatory-approved therapeutic dosing and pharmacodynamics windows and can be implemented instantly. From a regulatory authorities' perspective, the use of these systems may be viewed as reminders for improve of patient's adherence (268).

Ongoing clinical trials (NCT03843697; NCT03747705) are evaluating the effects of these regimens in patients with inflammatory bowel disease who have lost their response to anti-TNFs, and in patients with epilepsy who have lost response to anti-epileptics. Data from these studies is expected to find improved response to medications when applying variability-based therapeutic regimens. While this type of variability may not always fit into an underlying personalized variability pattern, it is likely to provide improvement over the fixed regimens which underlie drug resistance.

In the second step, a closed-loop algorithm should be implemented, where inputs are based on clinical outcomes chosen to generate therapeutic regimens. This step requires regulatory approval, as it involves changes of dosing regimens outside approved ranges, based on subject's clinical response, while keeping dosages within pre-determined safety boundaries. The algorithm may prevent certain unwanted side effects, as it enables skipping doses, but should not provide dosages which might potentially induce drug complications (267).

In the third step, host and disease-related patterns of variability would be quantified in a personalized manner, and then implemented in a true-random number generator. This step is expected to further improve the response rate and sustainability of beneficial effects of therapies in patients with chronic diseases. It involves a process of continuous adaptation of algorithm output to inputs from quantifiable variability parameters. These will be parameters associated with disease pathogenesis, host response, and the mechanism of action of the drug. For example, heart rate variability parameters can be used an input for generating therapeutic regimens for patients with heart failure; and measuring variabilities in cytokine response will help treat patients with immune disorders (267).

For all steps, genotypic and phenotypic parameters, which may impact algorithm output, are “ignored” by the platform. The algorithm continuously adapts itself to a clinically meaningful output as its sole endpoint. The sums of effects of all potential factors on a clinical endpoint are thus being considered without splitting them into individual variables. This contrasts with attempts made by most first-generation AIs to dissect and quantify only some of these parameters. Thus, the second-generation medical AI will not consider many unquantifiable measures, or use fixed rules which are based on artificial parameters, or on fitting of data from large datasets. Instead, the proposed platform adapts itself to a sum of all parameters via their effects on a sole measured endpoint in a single subject. The continually individualized dynamic and unpredictable changes in disease, host response, and environmental triggers will be accounted for via their effects on clinical outcomes, generating a dynamic output by the algorithm.

In the fourth step, an insightful database would be generated by the algorithm. Outputs from this database and from conventional datasets would then be used to introduce additional variables into the algorithm. The effects of these interventions would be continuously measured by evaluating their effect on a clinical endpoint in an individual subject (267).

**Figure 1:**
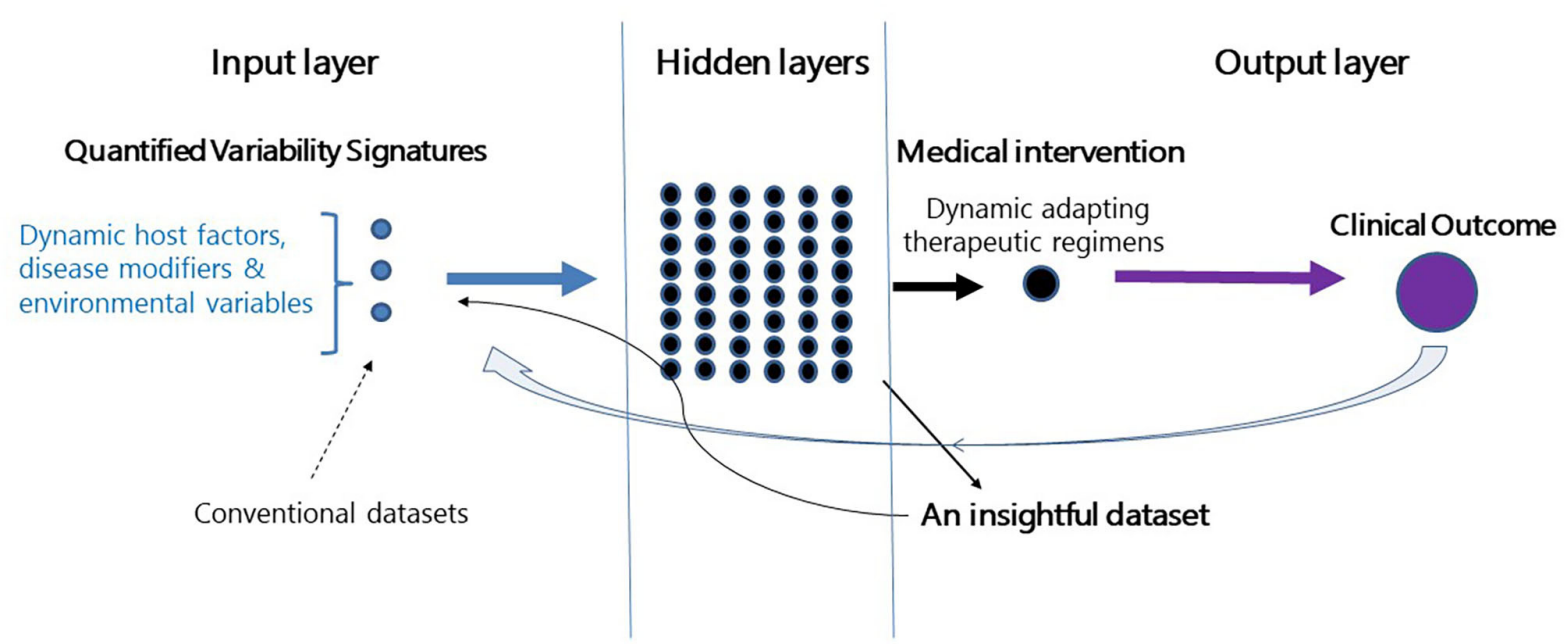
Establishment of a second-generation AI platform. Depiction of a closed-loop platform where a patient with a chronic disease is placed at the center. The closed feedback loop is responsive to the effect of therapy on clinical outcomes. The algorithm adapts itself dynamically to the effects of therapy on the endpoint. The algorithm quantifies personalized variability patterns and implements them into therapeutic regimens. In parallel, the algorithm generates an insightful database which evolves from analyzing outputs on the endpoint. The dataset collects the relevant variability-based quantifiable parameters which are associated with improved clinical outcomes. The dataset is continuously being updated based on the clinical response of each of the treated subjects.

Figure 1 shows a schematic presentation of a closed-loop algorithm where a single subject with a chronic disease is at the focus. A feedback loop is responsive to the effect of therapy on a clinically meaningful endpoint. The algorithm aims at this endpoint and adapts itself in a dynamic manner. The AI platform then quantifies personalized variability patterns relevant to the disease, host response, and environment-related variables. It combines these variables into a dynamic therapeutic regimen. In parallel, the algorithm generates its own insightful database which evolves from analyzing its outputs on organ function (160, 166).

All of this notwithstanding, doctors will most likely not be entirely replaced by AI systems. The platforms discussed here are expected to support physicians for improving patients' management (149, 269). While first-generation systems were designed to assist medical decisions, their penetration into clinical workflow has been limited. Second-generation systems are and will be designed to overcome some of the obstacles experienced over recent years. These new platforms should support better interactions between clinicians and algorithms, showing that a combination of human and AI outperforms either alone, and aiming at improvements to clinical outcomes. The dataset collects the variability-based quantifiable parameters which are associated with improved clinical outcomes. It continuously updated in accordance with the clinical response of each of the treated subjects (10, 270).

The platform described herein introduces a novel method wherein the focus of an algorithm is on improving clinical outcomes of individual subjects. It implements an *n* = 1 concept into a personalized therapeutic regimen by introducing individualized variability signatures into an algorithm. The system is based on improving the beneficial effects of therapeutic interventions. This makes it more likely to gain adherence from both patients and healthcare providers. The platform is designed to overcome compensatory mechanisms associated with drug resistance and disease progression, to ensure sustainable beneficial effects from medications. Results of ongoing clinical trials and future prospective studies are expected to enable further development of these platforms.

## Author Contributions

The author confirms being the sole contributor of this work and has approved it for publication.

## Conflict of Interest

YI is the founder of Oberon Sciences and is a consultant for Teva, ENZO, Protalix, Betalin Therapeutics, Immuron, SciM, Natural Shield, and Tiziana.

## Abbreviations

AI: artificial intelligence
EMR: electronic medical records
ML: machine learning
RCT: randomized controlled trials
NLP: natural language processing
TNF: tumor necrosis alpha
ConA: concanavalin A.
